# Genome wide association and genomic prediction for growth traits in juvenile farmed Atlantic salmon using a high density SNP array

**DOI:** 10.1186/s12864-015-2117-9

**Published:** 2015-11-18

**Authors:** Hsin-Yuan Tsai, Alastair Hamilton, Alan E. Tinch, Derrick R. Guy, Karim Gharbi, Michael J. Stear, Oswald Matika, Steve C. Bishop, Ross D. Houston

**Affiliations:** The Roslin Institute and Royal (Dick) School of Veterinary Studies, The University of Edinburgh, Midlothian EH25 9RG, Edinburgh, UK; Landcatch Natural Selection Ltd., 15 Beta Centre, Stirling University Innovation Park, Stirling, FK9 4NF UK; Edinburgh Genomics, Ashworth Laboratories, King’s Buildings, The University of Edinburgh, Edinburgh, EH9 3JT UK; Institute of Biodiversity, Animal Health & Comparative Medicine, University of Glasgow, Bearsden Road, Glasgow, G61 1QH UK

**Keywords:** Atlantic salmon, GWAS, Genomic-wide association analysis, Genomic prediction, Growth

## Abstract

**Background:**

The genetic architecture of complex traits in farmed animal populations is of interest from a scientific and practical perspective. The use of genetic markers to predict the genetic merit (breeding values) of individuals is commonplace in modern farm animal breeding schemes. Recently, high density SNP arrays have become available for Atlantic salmon, which facilitates genomic prediction and association studies using genome-wide markers and economically important traits. The aims of this study were (i) to use a high density SNP array to investigate the genetic architecture of weight and length in juvenile Atlantic salmon; (ii) to assess the utility of genomic prediction for these traits, including testing different marker densities; (iii) to identify potential candidate genes underpinning variation in early growth.

**Results:**

A pedigreed population of farmed Atlantic salmon (*n* = 622) were measured for weight and length traits at one year of age, and genotyped for 111,908 segregating SNP markers using a high density SNP array. The heritability of both traits was estimated using pedigree and genomic relationship matrices, and was comparable at around 0.5 and 0.6 respectively. The results of the GWA analysis pointed to a polygenic genetic architecture, with no SNPs surpassing the genome-wide significance threshold, and one SNP associated with length at the chromosome-wide level. SNPs surpassing an arbitrary threshold of significance (*P* < 0.005, ~ top 0.5 % of markers) were aligned to an Atlantic salmon reference transcriptome, identifying 109 SNPs in transcribed regions that were annotated by alignment to human, mouse and zebrafish protein databases. Prediction of breeding values was more accurate when applying genomic (GBLUP) than pedigree (PBLUP) relationship matrices (accuracy ~ 0.7 and 0.58 respectively) and 5,000 SNPs were sufficient for obtaining this accuracy increase over PBLUP in this specific population.

**Conclusions:**

The high density SNP array can effectively capture the additive genetic variation in complex traits. However, the traits of weight and length both appear to be very polygenic with only one SNP surpassing the chromosome-wide threshold. Genomic prediction using the array is effective, leading to an improvement in accuracy compared to pedigree methods, and this improvement can be achieved with only a small subset of the markers in this population. The results have practical relevance for genomic selection in salmon and may also provide insight into variation in the identified genes underpinning body growth and development in salmonid species.

**Electronic supplementary material:**

The online version of this article (doi:10.1186/s12864-015-2117-9) contains supplementary material, which is available to authorized users.

## Background

Atlantic salmon (*Salmo salar*), an anadromous species found primarily in the northern Atlantic Ocean, is widely known for its importance in both wild fishing and aquaculture. According to statistics from the Food and Agriculture Organization (FAO), the estimated global economic value of this species in 2010 was approximately $7.8 billion [1]. Atlantic salmon is also a model for genomic studies of salmonid species with extensive genomic resources and a recent availability of an assembled reference genome sequence [2]. Atlantic salmon breeding programs are the most advanced of all aquaculture species and routinely incorporate genomic information to construct pedigrees, and to improve selection accuracy via marker-assisted or genomic selection [3].

Genome-wide association studies (GWAS) are employed to assess the association between DNA sequence variants (typically SNPs) dispersed throughout the genome and complex traits of interest. To date, abundant GWAS have been conducted on human [4] and terrestrial livestock species [5, 6], resulting in the discovery of several genes and underlying mutations affecting traits of medical and economic importance. However, despite the contribution of GWAS to terrestrial livestock and human medical research, relatively few GWAS have been undertaken in aquaculture species to date, and have typically utilized relatively sparse SNP chips [7–9]. Recently, a high density publicly available SNP chip containing ~132 K verified SNP markers was developed [10] and gives the opportunity to apply GWAS at a resolution previously not possible in salmon.

Commercially important traits for salmon farming such as growth and disease resistance are a major focus for scientific research, with several QTL mapping studies performed for growth performance (e.g. [11–13]) and disease resistance (summarized in [14]). Studies of the genetic basis of growth related traits using QTL linkage mapping identified chromosome regions of interest; however, there is a lack of consistency between the location of the QTL in different populations [11, 13, 15]. Potentially, GWAS may be able to address some of the drawbacks of QTL mapping, such as the possible omission of QTL due to inadequate marker density [16]. Additionally, since GWAS detects SNPs in population-wide linkage disequilibrium with QTL affecting the trait, the potential for applying these markers directly in selective breeding is greater. While single marker-assisted selection is of limited value for polygenic traits, genomic estimated breeding values (GEBVs) can be calculated for candidate breeding animals using marker data, even in the absence of trait and/or pedigree information [17]. Studies using simulated data have shown the accuracy of prediction of breeding values using genomic data was significantly higher than using pedigree records alone [18, 19]. Few studies of genomic prediction using real data have been performed in aquaculture species, although one recent analysis of a recently admixed farmed Atlantic salmon population suggests that a genomic prediction approach can be effective at improving the accuracy of selection compared to pedigree records alone [20].

The objectives of this study were (i) to use the high density (~132 K) SNP array to estimate genetic parameters for weight and length of juvenile farmed salmon and compare to those based on pedigree; (ii) to detect individual SNPs/chromosomes associated with these traits; (iii) to estimate breeding values and prediction accuracy for the two traits by applying the pedigree and the genomic relationship matrix across different marker densities; (iv) to identify putative growth candidate genes by annotating the most significant markers from transcribed regions of the genome.

## Results

### Summary statistics and heritability

The final dataset used for the GWAS consisted of ~ 112 K QC-filtered SNPs successfully genotyped in 622 fish (from 61 full sibling families) with weight and length measurements taken approximately 1 year post-hatching. Sex of the fish was predicted based on the Y-specific probes on the SNP array (as described in Houston et al. [10]) and the population was evenly split between males and females (1:1.03). The weight and length traits were highly correlated at the phenotypic and genetic level (r ~ 0.96 in both). The overall heritability for both traits, as estimated by the genomic relationship matrix was ~ 0.6, compared to ~ 0.5 using the pedigree relationship matrix (Table 1).

**Table 1:** The heritability and summary statistics of the weight and length phenotypes

	Weight (g)	Length (mm)
Mean (std dev)	112.0 (24.0)	214.1 (16.1)
Heritability^a^ (std err ):		
G-matrix	0.60 (0.07)	0.61 (0.07)
A-matrix	0.48 (0.10)	0.51 (0.11)

^a^Heritability was estimated by the genomic relationship matrix (G-matrix) and pedigree-based relationship matrix (A-matrix) respectively

### Genome-wide association analysis

To determine which individual SNPs were associated with weight and length, a GWAS was performed on all markers. No SNPs reached the genome-wide significance level (using the stringent Bonferroni correction), whereas one SNP mapping to chromosome 17 surpassed the chromosome-wide significance level for length and was estimated to explain ~ 7 % of the additive genetic variation (Table 2). 684 of the 111,908 SNPs surpassed an (arbitrary) relaxed threshold [nominal *P* < 0.005 from model (1)] and were used for determining putative candidate genes (Additional file 1: Appendix 1 and Table 3). The p-value, allele frequency, additive and dominance effect, and proportion of additive genetic variance due to the SNP for the top ten markers for weight and length are given in Table 2. Full lists of the SNPs surpassing the relaxed threshold are given in Additional file 2: Appendix 2. The proportion of genetic variation explained by the top ten markers ranged between 0.003 to 0.12. Approximately 40 K SNPs had been assigned to corresponding chromosome using sire-based linkage mapping performed by Crimap software [21] as described in Houston et al. [10] and using the reference genome sequence (AKGD00000000.4). The quantile-quantile (Q-Q) plots generated using model (1) in the GWA analysis for weight and length are given in Additional file 3: Appendix 3.

**Table 2:** The p-value, allele frequency, additive (α) and dominance (δ) effect, and proportion of additive genetic variance explained for the top ten SNP markers associated with weight and length

Weight							
Marker	P-value	Allele frequency		Additive effect (s.e.)	Dominance effect (s.e.)	PVE	Chromosome
		p	q				(Unknown: n/a)
^a^AX87944147	2.8 e-05	0.69	0.31	4.97 (1.88)	8.76 (2.09)	0.003	n/a
^a^AX87934338	6.4 e-05	0.61	0.39	7.22 (2.00)	3.22 (2.08)	0.08	16
AX87992121	9.5 e-05	0.54	0.46	7.55 (1.97)	0.18 (2.11)	0.08	n/a
AX87888225	1.0 e-04	0.94	0.06	7.00 (6.28)	23.83 (6.66)	0.06	n/a
AX87943138	1.2 e-04	0.69	0.31	8.34 (2.07)	2.65 (2.29)	0.10	21
AX88223695	1.2 e-04	0.80	0.20	3.32 (2.76)	16.54 (3.02)	0.04	28
AX87959413	1.3 e-04	0.58	0.42	7.34 (1.81)	3.61 (1.96)	0.08	28
AX88127533	1.4 e-04	0.59	0.41	7.43 (1.84)	2.71 (1.98)	0.07	28
^a^AX87963258	1.4 e-04	0.57	0.43	5.80 (1.47)	2.00 (2.04)	0.05	17
AX88282141	1.5 e-04	0.56	0.44	6.68 (1.77)	0.56 (1.96)	0.07	21
Length							
Marker	P-value	Allele frequency		Additive effect (s.e.)	Dominance effect (s.e.)	PVE	Chromosome
		p	q				(Unknown: n/a)
^**a**^ **AX87963258**	**1.7 e-05**	**0.57**	**0.43**	**4.42 (0.99)**	**1.27 (1.37)**	**0.07**	17
AX88141678	5.3 e-05	0.77	0.23	6.84 (1.88)	1.74 (1.98)	0.07	5
^a^AX87944147	5.4 e-05	0.69	0.31	3.19 (1.27)	5.77 (1.40)	0.003	n/a
^a^AX87934338	7.3 e-05	0.61	0.39	4.91 (1.34)	1.71 (1.40)	0.08	16
AX87959512	9.1 e-05	0.68	0.32	5.46 (1.48)	0.21 (1.55)	0.08	20
AX88083269	1.0 e-04	0.59	0.41	4.76 (1.16)	1.99 (1.40)	0.08	n/a
AX88089073	1.6 e-04	0.70	0.30	4.77 (1.62)	1.07 (1.65)	0.05	20
AX88048182	1.6 e-04	0.78	0.22	6.65 (1.88)	1.96 (2.00)	0.12	5
AX88267406	1.6 e-04	0.78	0.22	6.65 (1.88)	1.96 (2.00)	0.12	5
AX88287764	1.7 e-04	0.85	0.15	3.33 (3.38)	12.33 (3.47)	0.04	n/a

Bold: chromosome-wide significance (*p* < 0.05)

^a^SNP appears in the lists for both traits.

**Table 3:** Summary of the putative homologous genes associated with SNPs surpassing the relaxed threshold (*P* < 0.005), the associated SNP name and predicted chromosome location on the salmon genome. The details of corresponding transcript id and SNP effect are given in Additional file 2: Appendix 2 and Additional file 1: Appendix 1

Marker ID	Gene	Chromosome^a^	Reference species
AX88089073	*POMT1*	20	Human/Mouse/Zebrafish
AX87884170	*MYH9*	03	Human/Mouse/Zebrafish
AX88052896	*GAPDH (GAPDHS)*	05	Human/Mouse/Zebrafish
AX87900517	*NOTCH3*	06	Human/Mouse/Zebrafish
AX88070408	*WDR35*	01	Human/Mouse/Zebrafish
AX88276725	*WDR35*	01	Human/Mouse/Zebrafish
AX88067081	*AGRN*	15	Human/Mouse/Zebrafish
AX87963258^b^	*RAI2*	17	Human/Mouse
AX87914686	*KNDC1*	01	Human/Mouse
AX87934385	*TXNRD3*	12	Human/Mouse
AX87906812	*ARHGEF7*	16 / 17	Human/Zebrafish
AX88009559	*DLG5*	01	Human/Zebrafish
AX87895800	*KLHL42*	17	Human/Zebrafish
AX87913460	*GUCY2F*	13	Human
AX87934385	*TXNRD1*	12	Zebrafish
AX88060914	*MYO18AB*	20	Zebrafish
AX87883353	*SYTL5*	21	Zebrafish
AX87913460	*GC3*	13	Zebrafish
AX88168740	*SI:CH211-181D7.1*	03	Zebrafish
AX88009559	*DLG5A*	01	Zebrafish
AX88254864	*PGBD4(5 OF 8)*	02	Zebrafish
AX88049616	*PGBD4(5 OF 8)*	02	Zebrafish

^a^Corresponding chromosome was based on the Atlantic salmon reference genome (AKGD00000000.4) and the chromosome assignments given in Houston et al. [10], see methods for additional details

^b^Chromosome-wide significance

*AGRN*: agrin; *ARHGEF7*: Rho guanine nucleotide exchange factor (GEF) 7; *GAPDH*: Glyceraldehyde-3-Phosphate Dehydrogenase; *DLG5*: Discs, Large Homolog 5 (Drosophila); *RAI2*: Retinoic acid-induced protein 2; *KNDC1*: Kinase Non-Catalytic C-Lobe Domain (KIND) Containing 1; *GUCY2F*: Guanylate Cyclase 2 F, Retinal; *POMT1*: Protein-O-Mannosyltransferase 1; *GC3*: guanylate cyclase 2D, membrane (retina-specific); *KLHL42*: kelch-like family member 42; *TXNRD1*: Thioredoxin Reductase 1; *TXNRD3*: Thioredoxin Reductase 3; *WDR35*: WD repeat domain 35; *MYH9*: myosin, heavy chain 9, non-muscle*; NOTCH3*: notch 3; *MYO18AB*: myo18ab; *SYTL5*: synaptotagmin-like 5

### Genomic prediction within population

**Fig. 1:**
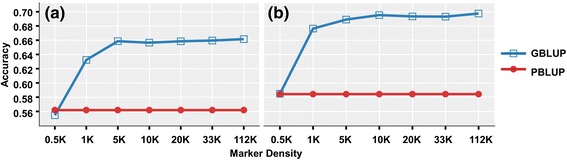
The estimated prediction accuracy of the (**a**) length and (**b**) weight traits when applying GBLUP and PBLUP across different marker densities (from 0.5 K to 112 K SNPs)

The use of the SNP markers for genomic prediction (GBLUP) of the weight and length traits was assessed and compared to the equivalent model using the pedigree to define relationships between the animals (PBLUP) using a five-fold cross validation design. The accuracy of the GBLUP model was approximately 20 % higher than PBLUP for both traits when using all markers in the model, reaching a value of approximately 0.7 within this population. Interestingly, while the prediction accuracy was improved by approximately 20 % with increased marker density from 0.5 K to 5 K SNPs, there was very little or no improvement in accuracy of prediction with increased marker density beyond this level. At the lowest marker density analyzed (0.5 K), the accuracy of GBLUP and PBLUP had the similar performance in both traits (Fig. 1). However, it should be noted that the training and validation populations used for this analysis will contain closely related animals.

### Putative gene identification

A large proportion of the SNPs on the 132 K Axiom array were derived from an RNA-Seq experiment and, therefore, are likely to be located within genes. 109 of the 684 SNPs surpassing a nominal significance threshold were matched with salmon fry transcriptome data using blastn alignment. From these 109 transcripts, twelve, seven, and fifteen corresponding unique peptides were identified from human, mouse, and zebrafish database respectively using strict alignment criteria (E ≃ 0). Five genes were identified in all reference species, while ten, seven, and two genes were detected specifically in the zebrafish, human, and mouse databases respectively. Details including the associated gene name, putative chromosome in Atlantic salmon, gene ontology (GO), transcript id and gene id are given in Additional file 1: Appendix 1. Summaries of the identified genes are given in Table 3 while the effects associated with these genetic markers are given in Additional file 2: Appendix 2.

The single marker that surpassed the chromosome-wide significance level for length (and also appears to have similar association with weight; Table 1) was annotated as Retinoic acid-induced protein 2 (*RIA2*; Table 3). Retinoic acid is a critical regulator of development, cellular growth, and differentiation [22] although the specific role of this RA induced gene is unknown.

## Discussion

### Genome wide association study

A high density SNP array [10] was applied to estimate genetic parameters and map SNPs associated with early growth of farmed salmon, as reflected by weight and length measurements at 1 year of age. The estimates of trait heritability when using the genomic relationship matrix was comparable but slightly higher than the equivalent model using the pedigree relationships (~ 0.6 vs ~ 0.5). While no SNPs surpassed the stringent genome-wide significance threshold, one SNP surpassed the chromosome-wide threshold for length (*p* < 0.05). Therefore, the GWAS results suggest that early growth in salmon is highly heritable but with a polygenic architecture and no evidence for major QTL. Based on previous linkage mapping and the current salmon reference genome assembly (AKGD00000000.4), the individual SNPs with the lowest P value for the growth traits were located on chr. 5, 16, 17, 20, 21 and 28. QTL associated with growth traits have been reported on the same chromosomes in some (but not all) previous studies in Atlantic salmon (eg. [11–13, 15]). The proportion of variance explained (PVE) by each individual marker was relatively small (up to 12 %) for the growth traits. The data in the current study support previous studies suggesting that there is a lack of consistent, cross-population, major QTL affecting growth in Atlantic salmon. Previous studies have performed GWA analyses to identify genetic variants associated with complex traits such as flesh texture, fat content and sexual maturation by using a ~ 6 K SNP array in farmed Atlantic salmon [7, 9]. In the current study, we used a substantially higher density of SNPs (~ 112 K), which may have facilitated the detection of QTL in regions not covered by previous lower density SNP platforms.

### Assessment of genomic prediction

Due to practical and financial limitations, such as the previous lack of a high density genotyping platform, relatively few studies into genomic prediction have been undertaken using real data in aquaculture species. A recent study by Odegard et al. [20] showed prediction accuracies of 0.34 and 0.36 for the traits of sea lice resistance and fillet colour respectively when using PBLUP, whereas GBLUP improved the accuracies by 32 % to 51 % for sea lice resistance, and up to 22 % for fillet colour. Previous studies applying simulated data have also indicated that GBLUP would have significantly higher accuracy compared to the equivalent model using pedigree records in the typical half/full-sibling family structure of salmon breeding programs (eg. [20, 23]). Our results also show that the BLUP model applying genomic data had higher accuracy than using pedigree information for both the weight and length traits, with an improvement of approximately 20 % to values close to 0.7. This is promising for the application of genomic prediction within salmon breeding programs, where it may be most effective for traits evaluated in siblings or other close relations of the selection candidates.

It is also noteworthy that ~ 5000 high quality informative SNPs are sufficient to achieve this increase in prediction accuracy in this population. Genotyping and field data collection are costly and the relative advantage of using SNP data in selection depends on these costs versus the value of the extra improvement in the traits of interest. Therefore, while the high density SNP array is more than adequate for within-population genomic prediction, the use of a cheaper and lower density SNP platform is likely to be most cost-effective. The cost-benefit is also likely to be most favourable for traits with high economic value and that cannot be measured directly on the selection candidates (e.g. disease resistance or fillet quality traits). However, it is important to note that (i) this is a relatively small dataset for assessing genomic prediction and (ii) the training and validation population will contain closely related animals. As such, genomic prediction in this dataset will be based on both linkage and linkage disequilibrium information, which is likely to result in increased accuracy of prediction and reduced need for high density markers compared to scenarios where the training and validation populations are distantly related to each other. It is plausible that with more distant relationships between the populations, a higher marker density and larger sample size would be required to achieve improvements in selection accuracy over traditional BLUP. Further, the high levels of linkage disequilibrium will result in greater power to detect QTL via GWAS, but lower resolution to estimate QTL position. Simulation studies are generally consistent with the results based on real data presented in the current study: Vela-Avitua et al. [23] reported that the prediction accuracy using sparse genomic data was equivalent or lower than using the classical pedigree model with sparse markers (10 – 20 SNPs/M) across traits with different heritabilities (h^2^ ~ 0.1, 0.3 and 0.8), while Hickey et al. [24] demonstrated that increasing marker density above ~ 10 K results in little or no improvement in prediction accuracy in maize populations, while the results of Gorjanc et al. [25] also show only minor increases in accuracy above this level in simulated livestock datasets. Finally, Odegard et al. [20] detected little increase in accuracy with increases in marker density above 22 K for fillet colour or lice resistance in a commercial salmon population.

### Putative gene identification

The single SNP exceeding the chromosome-wide significance level for length was mapped to chr.17, and its predicted location is within the retinoic acid-induced protein 2 gene (*RAI2*). Although the function of *RAI2* is not yet clear, this gene is suggested to be involved in the control of cellular growth and embryo development [26]. Retinoic acid is well established as a key regulator of growth and differentiation in early life [22], and is involved in the regulation of bone formation and mineralization in salmon [27]. Therefore, *RAI2* can be considered both a positional and a biological candidate for an effect on regulation of growth in juvenile salmon. Genes associated with the other markers discovered surpassing the arbitrary relaxed significance threshold (*P* < 0.005) were also identified by aligning with human, mouse, and zebrafish databases (Table 3). Amongst these was a SNP in *POMT1* (Protein-O-Mannosyltransferase 1) which produces the *POMT* enzyme complex, dysregulation of which can contribute to the formation of abnormal basement membranes, which can lead to muscular dystrophy [28]. Interestingly, the *AGRN* (agrin) gene also appears to have a key regulatory role in basement membranes of neuromuscular junctions, and is involved in the inhibition, storage, activation of varied growth factors [29], clustering of voltage-gated sodium channels, and G-protein coupled acetylcholine receptor signaling pathway [30], all of which are essential for fundamental cell development. In addition, *NOTCH3* (notch 3) and the *NOTCH3* receptor have critical roles in the development and maintenance of vascular smooth muscle cells [31, 32]. Finally, genes associated with ATP binding and motor activity, such as *MYH9* (myosin, heavy chain 9) and *MYO18AB* were also identified amongst the nominally significant markers. While a proportion of the nominally significant SNPs (and therefore the genes identified) will clearly be false positives, highlighting these genes provides the opportunity to cross-reference with future studies to identify with higher confidence the putative candidates underlying growth in salmon.

## Conclusions

The results of the current study show that early growth traits are highly heritable in farmed Atlantic salmon, and that the heritability can be estimated by using either the genomic or the pedigree relationship matrix. The GWA analysis showed that there are likely to be small effect QTL on several chromosomes, but there was no evidence for major QTL and these traits appear to be highly polygenic in nature. A SNP in the retinoic acid-induced protein 2 gene on chromosome 17 reached chromosome-wide significance, and is a plausible positional and functional candidate gene. Other genes identified from nominally significant SNPs will be useful for cross-referencing with similar studies in salmon and may form candidates for follow up studies to assess their function in regulation of growth in salmon. For breeding value prediction using genomic and pedigree data, GBLUP had better accuracy than PBLUP in general with accuracy of ~ 0.7 attained for early growth traits using GBLUP in this population. As few as 5 K SNPs gives close to maximal accuracy within population, suggesting that only moderate marker density is likely to be suitable for GS breeding programs for similar highly heritable but polygenic traits where the discovery populations have close relationships with the selection candidates. However, it is important to note that increased marker density is likely to be advantageous, alongside larger sample size, when attempting to predict genomic breeding values in more distantly related animals.

## Methods

### Ethics statement

All animals were reared in accordance with the U.K. Home Office regulations regarding the use of animals in experiments. The trial was carried out at the Marine Environmental Research Laboratory (Machrihanish, UK) and approved by the ethical review committee in University of Stirling (Stirling, UK). Fish were purchased from Landcatch which are accredited participants in the RSPCA Freedom Foods standard, the Scottish Salmon Producers Organization Code of Good Practice, and the EU Code-EFABAR Code of Good Practice for Farm Animal Breeding and Reproduction Organizations.

### Animals and phenotype measurement

The population used in the current study was a subset of those described in Gharbi et al. [33]. Briefly, eggs from the 2007 cohort of Landcatch Natural Selection (LNS, Ormsary, UK) broodstock fish were hatched and reared in separate family tanks in freshwater. At the post-smolt stage, fish were transferred to sea water environment (Machrihanish, UK). The one-year-old post-hatch fish from 62 full sibling families were PIT-tagged and transferred to a single tank. All fish were measured for body weight (g) and body length (mm). Parents and offspring of families represented by a minimum of 6 fish in the population (712 fish from 61 full sibling families) were selected for genotyping. The PIT tags were used to assign offspring to parents and construct the pedigree.

### SNP array genotyping

DNA from the 712 fish was extracted using the DNeasy-96 tissue DNA extraction kits (Qiagen, Crawley, UK) and then genotyped for the Affymetrix Axiom SNP array containing ~132 K validated SNPs [10] (http://www.affymetrix.com/support/technical/datasheets/axiom_salmon_genotyping_array_datasheet.pdf). Starting with these validated SNPs, filtering of SNP data was performed using the Plink software [34] to remove individuals and SNPs with excessive (> 1 %) Mendelian errors and SNPs with minor allele frequency (MAF) < 0.05 in this dataset. A total of 111,908 remaining SNPs were retained for 622 fish (534 offspring, 28 sires and 60 dams). The phenotypic sex of the offspring was unknown and, therefore, the Y-specific probes on the array were used to predict the genetic sex of the fish based on the putative sex determining gene [35], as described in Houston et al. [10].

### Statistical analysis

#### Heritability estimation

Genetic parameters for the weight and length traits were tested fitting animal as a random effect. The estimation was performed using a REML analysis assuming the following model:

1 $$ \mathbf{y}=\mathbf{X}\mathbf{b}+\mathbf{Z}\mathbf{u}+\mathbf{e} $$

where **y** is the observed trait, **b** is the fixed effect of sex, **u** is the vector of additive genetic effects, **e** is the residual error and **X** and **Z** the corresponding incidence matrices for fixed effects and additive effects, respectively. The covariance structure for the genetic effect was calculated either using pedigree (**A**) or genomic (**G**) information (i.e. **u** ~ N(0, Aσ_a_^2^) or N(0, Gσ_a_^2^)). Hence, the narrow sense of heritability was estimated by the additive genetic variance and total phenotypic variance, equaling to:

2 $$ {h}^2={\sigma^2}_a\kern0.24em /\;{\sigma^2}_p $$

where σ^2^_a_ is the additive genetic variance and σ^2^_p_ is the total phenotypic variance which is a sum of *σ*^*2*^_*a*_ *+ σ*^*2*^_*e*_.

The analysis was implemented using the ASReml 3.0 software [36]. The genomic relationship required for the analysis was calculated using the Genabel ‘R’ package [37] and method of VanRaden [38], and then inverted applying the standard ‘R’ function.

#### Genome-wide association study

The GWAS was performed using the two-step GRAMMAR method implemented in Genabel [37]. Firstly, the trait data were corrected for the fixed effect and polygenic effects (fitting the genomic relationship matrix) using model (1) above. Secondly, the association between the individual SNPs and the residuals from model (1) was applied using the ‘mmscore’ method [39]. The genome-wide and chromosome-wide significance thresholds were determined by Bonferroni correction (0.05/N), where N represents the number of QC-filtered SNPs across the entire genome (genome-wide) and on each chromosome (chromosome-wide).

Subsequently the allelic substitution effects of SNPs from the GWA analysis surpassing an arbitrary relaxed threshold (*P* < 0.005, ~ top 0.5 % of all markers) were estimated using ASReml 3.0 [36] fitting the mixed model (1) as previously described plus the SNP as the fixed effects.

The SNP additive effect (*α*) was calculated as half the difference between the predicted phenotypic means of the two homozygotes, (AA-BB)/2, and the dominance effect (*δ*) was AB – [(AA + BB)/2], where the AB represents the predicted phenotypic mean of the heterozygote. The proportion of genetic variance explained (PVE) by the SNP was estimated using the following equation:

3 $$ PVE=\left[2pq\;{\left(a+\delta\;\left(q-p\right)\right)}^2\right]\;/\;{V}_A $$

where *α* and *δ* are the additive and dominance effect respectively, the p is the frequency of the major allele and q is the frequency of the minor allele, and V_A_ is the total additive genetic variance of the trait obtained when no SNP effects are included in the model. For certain markers containing two genotypes, the dominance effect (*δ*) was not included in the equation (Additional file 2: Appendix 2).

#### Genomic prediction

Estimated breeding values were obtained using the pedigree relationship (PBLUP) or the genomic one (GBLUP). These predictions were compared in terms of their accuracy to predict an unknown phenotype. In order to do so, a five-fold cross validation analysis was performed using the individuals with genotype data and phenotypes in both traits.

The individuals were randomly divided into five non-overlapping subsets (i.e. each subset contains one fifth of the data corresponding to ~ 106 individuals). One subset of data was then used as a validation set and the reminder of the data [four fifths (*n* ~ 425)] was used as the training population. The phenotype recorded in the validation population was then masked and breeding values were estimated using ASReml 3.0 assuming model (1). Accuracy was calculated as the correlation between the predicted EBVs of the validation set and the actual phenotypes divided by the square root of the heritability [*~ r(y*_*1*_*,y*_*2*_*)/h*] using all individuals. The whole procedure was independently replicated five times and average accuracy values were calculated.

#### Comparison of different SNP densities

We compared the use of different SNP marker densities of 0.5 K, 1 K, 5 K, 10 K, 20 K, 33 K, and 112 K (full dataset) for GEBV calculation. Firstly, as part of a pipeline for designing a lower density SNP genotyping platform for routine genomic evaluations, a subset of ~ 33 K SNPs were selected from the ~ 132 K array as follows: (i) only polymorphic high resolution SNPs were retained as defined using Affymetrix software, (ii) only one SNP per genome contig in the salmon genome assembly was retained (NCBI Assembly GCA_000233375.1), (iii) removed one of every pair of SNPs with r^2^ > 0.65 based on the Landcatch Natural Selection samples from the test plate of samples as described in Houston et al. [10], (iv) removed any remaining SNPs with a MAF < 0.1 and ‘missingness’ > 0.03 in the above samples and (v) added any SNPs not included above that reached a nominal significance threshold in a genome-wide association analysis for disease resistance (data not shown). From this reduced set of ~ 33 K SNPs, further subsets were taken at random to create SNP densities of 0.5 K, 1 K, 5 K, 10 K, and 20 K markers.

### Putative gene identification

Based on the result of the GWA analysis, the SNPs surpassing the relaxed significance threshold (*P* < 0.005 in model (1), ~ top 0.5 % of markers) were chosen to identify those located within or proximal to genes. Firstly, the flanking sequence of all the significant markers were aligned (using blastn) with an Atlantic salmon fry transcriptome database from RNA-seq of salmon fry in a separate study in which a large proportion of the SNPs on the array were discovered (described in Houston et al. [10]). Only markers whose flanking sequences that matched exactly with reference transcriptome database except at the SNP position was selected. These transcripts were used to align (using blastx) with human (*Homo sapiens*), mouse (*Mus musculus*), and zebrafish (*Danio rerio*) peptide reference database respectively (downloaded from http://www.ensembl.org/index.html; May 2014), from which a stringent criterion of e-value ≃ 0 were used as evidence for homology. Secondly, for each unique peptide in each of the species, the corresponding gene id, associated gene name, chromosome position, and gene ontology (GO) were retrieved from ensembl biomart database (retrieved from http://www.ensembl.org/biomart; *Jun.* 2014) respectively. The corresponding chromosome of SNP markers were identified by aligning the marker and its flanking sequence with salmon reference genome sequence (AKGD00000000.4) and existing LG mapping [10].

## Additional files

Additional file 1:
**Appendix 1.** List of identified putative gene name, chromosome position, gene ontology (GO), transcript id and gene id of three reference species databases. (XLSX 71 kb) Additional file 2:
**Appendix 2.** Summary of significant markers, their p-values, allele frequencies, additive and dominance effects, and proportion of genetic variance due to the SNPs, for weight and length respectively. (XLSX 21 kb) Additional file 3:
**Appendix 3.** The Q-Q plots of weight and length generated in GWA analysis. (XLSX 67 kb)

## Abbreviations

BLUP: best linear unbiased prediction
EBV: estimated breeding values
GBLUP: BLUP with a genomic relationship matrix
GEBV: genomic estimated breeding values
GS: genomic selection
GWAS: genome-wide association study
MAF: minor allele frequency
PBLUP: BLUP with a pedigree relationship matrix
PEBV: pedigree estimated breeding values
QTL: quantitative trait loci
SNP: single nucleotide polymorphism
